# Who is teaching and supervising our junior residents' central venous catheterizations?

**DOI:** 10.1186/1472-6920-11-16

**Published:** 2011-04-25

**Authors:** Irene WY Ma, Elise Teteris, James M Roberts, Maria Bacchus

**Affiliations:** 1 Division of General Internal Medicine, University of Calgary, Calgary, AB, Canada; 2 W21C, University of Calgary, Calgary, AB, Canada; 3 Division of General Internal Medicine, University of British Columbia, Vancouver, BC, Canada

## Abstract

**Background:**

The extent to which medical residents are involved in the teaching and supervision of medical procedures is unknown. This study aims to evaluate the teaching and supervision of junior residents in central venous catheterization (CVC) by resident-teachers.

**Methods:**

All PGY-1 internal medicine residents at two Canadian academic institutions were invited to complete a survey on their CVC experience, teaching, and supervision prior to their enrolment in a simulator CVC training curriculum.

**Results:**

Of the 69 eligible PGY-1 residents, 32 (46%) consenting participants were included in the study. There were no significant baseline differences between participants from the two institutions in terms of sex, number of ICU months completed, previous CVC training received, number of CVCs observed and performed. Only 16 participants (50%) received any CVC training at baseline. Of those who received any training, 63% were taught only by senior resident-teachers. A total of 81 CVCs were placed by 17 participants. Thirty-two CVCs (45%) were supervised by resident-teachers.

**Conclusions:**

Resident-teachers play a significant role both in the teaching and supervision of CVCs placed by junior residents. Educational efforts should focus on preparing residents for their role in teaching and supervision of procedures.

## Background

The ability to perform bedside procedural skills competently is an important part of medical practice. Procedures such as lumbar puncture, thoracentesis, and central venous catheterization (CVC), are commonly performed for diagnostic and/or therapeutic reasons and are often taught in residency training programs [1,2]. Indeed, for CVC, competency in this skill is a stated objective for a number of postgraduate medical training programs [3-7].

However, despite efforts in improving the training of technical skills [8], many residents are uncomfortable performing procedures [9]. Inadequate clinical exposure to procedures [2] and an insufficient supply of faculty proficient in teaching and supervising procedures have been previously cited as barriers to procedural training [1,10,11].

On the ward, resident teachers play an important role in the education of junior learners [12,13]. With respect to procedural supervision, it has recently been reported that many residents supervised procedures prior to feeling comfortable with performing the procedure themselves [14]. However, it is unknown to what extent do residents participate in the teaching and supervision of procedures.

Therefore, we aimed to evaluate the extent of teaching and supervision of CVC insertion by junior residents at two Canadian academic internal medicine residency programs. An estimation of the prevalence of resident-supervision and resident-teaching will help guide medical educators in terms of where to focus their curriculum efforts.

## Methods

During the academic year of 2009 (July 2009 to May 2010), all junior residents in their PGY-1 year from the University of British Columbia (UBC) (n = 47) and University of Calgary (n = 22) were invited to enroll in our simulation CVC educational curriculum. Details of this simulator curriculum have been previously described [15]. Only residents who provided written informed consent were included in this study. The study was approved by ethics review board from both academic institutions.

Prior to the start of the CVC course, which occurred throughout the academic year, participants were invited to complete an anonymous survey outlining their baseline CVC experience. In addition, they were asked to estimate the total number of and type of CVCs placed during their training, as well as information of who supervised those procedures. Supervision at both institutions is informally defined by the presence of a supervisor in the room where the procedure is being performed. The supervisor may or may not be gowned and gloved. However, a procedure performed without a supervisor directly in the room is considered an unsupervised procedure.

### Data Analysis

Baseline group comparisons were made with the use of Wilcoxon rank-sum tests and Fisher's exact tests where appropriate. Comparisons in proportions were made with the use of chi-square tests. All reported *P *values are two-sided. Analyses were performed using SAS statistical program version 9.1 (SAS Institute, Cary, NC).

## Results

A total of 32 participants were included in this study. Twenty-two participants out of 47 PGY-1 trainees (47%) were recruited from UBC and ten out 22 PGY-1 trainees (45%) were recruited from the University of Calgary. There were no significant baseline differences between the cohort from UBC and that from the University of Calgary (Additional file 1).

### Training and Supervision

Prior to the simulation training, only 16 (50%) participants had received any training on CVC (Additional file 1). Ten out of these 16 participants (63%) were taught only by senior resident-teachers. The remaining six participants were taught by faculty members only (n = 3), a combination of faculty and senior resident-teachers (n = 2), while one participant was taught by a resident of the same level of training as the participant (n = 1). Of the 16 participants who received prior CVC training, majority of the training was received within the context of patient care (n = 14, 88%). One participant received prior simulator training, while one participant received training in the context of patient care as well as on simulators.

**Figure 1:**
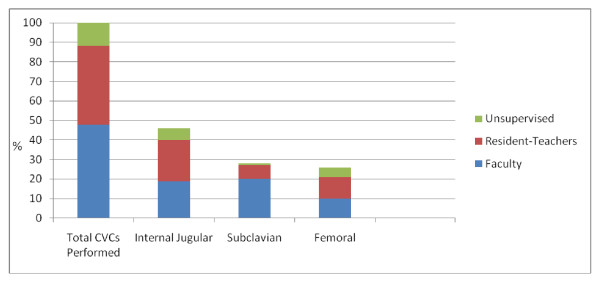
**Percentage of central venous catheters (CVC) supervised by faculty and resident-teachers**. Bar graph of percent of of central venous catheters supervised by faculty and resident-teachers.

At baseline, a total of 81CVCs had been placed by 17 PGY-1 participants. Of the 81 CVCs performed, 71 (88%) were supervised. Overall, 39 of the 81 CVCs (48%) were reported to be supervised by faculty members and 32 (40%) by residents (Figure 1). Ten of the 81 CVCs (12%), performed by three participants, were unsupervised. All three participants had previously placed more than nine CVCs.

### Supervision of CVCs Based on Line-type

Of the 81 CVCs placed, 37 were internal jugular lines, 23 were subclavian, and 21 were femoral lines (Figure 1). Faculty members supervised 70% of subclavian, 41% of internal jugular, and 38% of femoral lines. Indeed, faculty supervision is significantly higher for subclavian lines than for non-subclavian lines (*P *= 0.02).

## Discussion

The results of this study confirm that resident-teachers play an important role both in the teaching and supervision of CVCs for junior residents. Resident-teachers were responsible for teaching 63% of the junior residents who received any training, and much of this training occurred in the context of patient care. In terms of CVC supervision, 40% of all CVCs were supervised by residents. In contrast to subclavian CVCs, where faculty supervision was reported to occur 70% of the time, resident-teachers supervised more non-subclavian CVCs than faculty members (45% vs 40%). Lastly, only 12% of CVCs by junior residents were unsupervised, and each of the unsupervised CVCs was placed by trainees with considerable CVC experience.

A number of factors may potentially be responsible for significant involvement of residents in the teaching and supervision of CVCs placed by junior residents. First, over the past twenty years, the number of general internists who perform procedures has been shown to be declining [16]. By 2004 in the United States, only 16% of general internists reported performing CVC in their practice, compared with 39% in 1984 [16]. Not surprisingly, internists are increasingly less confident in their ability to teach and supervise procedures [10]. As faculty members become increasingly uncomfortable with teaching or supervising CVC, resident-teachers perhaps may be taking an increasing role in CVC teaching and supervision. Second potential reason behind high involvement of resident-teachers is that performances of CVCs may be occurring at times when faculty members are not as readily available. For example, CVCs placed at night may not have the same level of supervision as CVCs placed during the day [17]. Such discrepancies in supervision between weekdays and weekends/evenings have previously been reported for the supervision of running cardiac arrest resuscitations in teaching hospitals [18]. Third, having residents function as teachers or supervisors maybe mutually beneficial for both the senior resident-teacher and the junior resident-learner [19]. The very act of teaching may enhance learning on the part of the resident-teacher [20], while junior resident-learners may learn better from a teacher whose performance level is more similar to the learner's than from an expert [19,21]. Indeed, in a recent survey of medical residents, 86% of survey participants felt that medical procedures should be taught by a senior resident or fellow [11]. However, the disadvantage of placing resident-teachers, not yet comfortable with the procedure [14], in the position of supervising others may potentially result in adverse patient safety consequences. Complication rates of procedures supervised by resident-teachers, compared with those supervised by faculty, should be further evaluated. In a recent survey of internal medicine residents in California, up to 26% of PGY-2 residents reported supervising central venous catheterization (CVC) before feeling comfortable with their own procedural performance [14]. The observation that resident-teachers play a significant role in the teaching and supervision of CVC for junior trainees argues for the need for educational intervention for senior-resident-teachers. Indeed, a needs assessment of teaching skills for surgical residents has previously demonstrated that the item, "provide effective coaching with supervision of performance of technical procedures," was rated as the most important teaching activity by the survey participants [22]. What educational intervention best suits the needs for teaching procedural skills is unknown. In a systematic review of residents-as-teachers curricula, more than half of the reported interventions consisted of a one-off intervention [23]. A combination approach of including a longitudinal teaching program with a one-off workshop for teaching procedural skills has also been previously described [24]. Effectiveness of these educational interventions, however, remains unclear [23].

Our study has a number of limitations. First, the data was obtained by self-reporting measures and accuracy of the data was not verified. However, given that most of the residents (84%) have performed fewer than five CVCs, accuracy of the recalled information is likely higher than a comprehensive survey administered to residents at all levels of training. Second, results represent only on 46% of the combined PGY-1 population and may limit the generalizability of the conclusions. Specifically, our study reports 40% of CVCs were supervised by residents. Without capturing all CVCs placed by PGY-1s in both institutions, the degree of bias is unknown. However, the goal of our study was to evaluate the extent of resident supervision. While the percentage of CVC supervision may be subject to bias, a reported number of 32 CVCs in two academic institutions is likely the minimum number of CVCs being supervised by resident-teachers. This represents a conservative estimate of the extent of involvement by resident-teachers. Thirdly, despite the fact that both academic institutions in our study have a policy on having a faculty supervisor be available at all times for the trainees, whether or not faculty members were less likely to be called upon to supervise procedures outside the standard work hours than during standard work hours was not captured by our survey. Lastly, we did not survey the senior resident-teachers to ascertain their degree of comfort in supervising or teaching procedures. However, based on work by others [14], it is highly likely that a number of the senior resident-teachers would be uncomfortable with their role. Future study should focus on the clinical consequences of having resident-teachers supervise procedures, types of teaching skills required for teaching and supervising procedures, and how to optimize educational interventions for our resident-teachers.

## Conclusions

In conclusion, resident-teachers play a large role in the teaching and supervision of CVCs for junior residents. Educational efforts should focus on preparing residents for their role in teaching and supervision of procedures.

## Competing interests

The authors declare that they have no competing interests.

## Authors' contributions

IWYM: participated in research design, acquired, analyzed and interpreted data; drafted the paper. ET: participated in research design, acquired, analyzed data, and revised the paper critically. JMR: participated in research design and revised the paper critically. MB: participated in the interpretation of data, revised it critically. All authors read and approved the final manuscript.

## Pre-publication history

The pre-publication history for this paper can be accessed here:

http://www.biomedcentral.com/1472-6920/11/16/prepub

## Supplementary Material

Additional file 1 **Appendix A - Baseline Characteristics of Participants**. Table of baseline characteristics of participants. Click here for file
