# Supplementary material for: Who is teaching and supervising our junior residents' central venous catheterizations?
Source: BMC Med Educ. 2011 Apr 25;11:16. doi: 10.1186/1472-6920-11-16 (PMC3098212; doi:10.1186/1472-6920-11-16)
Supplement: Additional file 1 — Appendix A - Baseline Characteristics of Participants. Table of baseline characteristics of participants. [file 1472-6920-11-16-S1.DOC]

Appendix A. Baseline Characteristics of Participants

| Characteristic | University of  British Columbia | University of  Calgary | p-value |
| --- | --- | --- | --- |
| Number of participants | N=22 | N=10 |  |
| Sex |  |  |  |
| Male | 11 (50) | 6 (60) | 0.71 |
| Female | 11 (50) | 4 (40) |  |
| Number of ICU months completed |  |  |  |
| None | 13 (59) | 5 (50) | 0.49 |
| One | 7 (32) | 5 (50) |  |
| Two | 2 (9) | 0 (0) |  |
| Has received training on CVC | 12 (55) | 4 (40) | 0.70 |
| Of those who received training, who did the teaching? |  |  |  |
| Faculty member | 3 (25) | 2 (50) | 0.55 |
| Senior resident | 8 (67) | 4 (50) | 0.52 |
| A resident same level or more junior | 1 (8) | 0 (0) | 1.00 |
| Of those who received training, was training done on… |  |  |  |
| Patient | 11 (92) | 4 (100) | 1.00 |
| Simulator | 1 (8) | 1 (25) | 0.45 |
| Median total no. of CVC observed (range) | 5 (0-17) | 3 (0-20) | 0.47 |
| Median total no. of femoral CVC observed | 2 (0-5) | 1 (0-5) | 0.34 |
| Median total no. of IJ CVC observed | 2 (0-15) | 2 (0-10) | 0.95 |
| Median total no. of SC CVC observed | 0 (0-5) | 0 (0-5) | 0.84 |
| Median total no. of CVC performed | 1 (0-20) | 1 (0-10) | 0.86 |
| Median total no. of femoral CVC performed | 0 (0-8) | 0 (0-2) | 0.30 |
| Median total no. of IJ CVC performed | 0 (0-6) | 1 (0-5) | 0.72 |
| Median total no. of SC CVC performed | 0 (0-6) | 0 (0-6) | 0.73 |
